# Expenditure and resource utilisation for cervical screening in Australia

**DOI:** 10.1186/1472-6963-12-446

**Published:** 2012-12-05

**Authors:** Jie-Bin Lew, Kirsten Howard, Dorota Gertig, Megan Smith, Mark Clements, Carolyn Nickson, Ju-Fang Shi, Suzanne Dyer, Sarah Lord, Prudence Creighton, Yoon-Jung Kang, Jeffrey Tan, Karen Canfell

**Affiliations:** 1 Cancer Research Division, Cancer Council NSW, 153 Dowling Street, Woolloomooloo, Sydney, NSW, 2011, Australia; 2 Screening and Test Evaluation Program, School of Public Health, University of Sydney, Sydney, NSW, 2006, Australia; 3 Victorian Cytology Service, 752 Swanston Street, Carlton, Melbourne, VIC, 3053, Australia; 4 National Centre for Epidemiology and Population Health, Australian National University, Canberra, ACT, 2000, Australia; 5 Centre for Women’s Health, Gender and Society, University of Melbourne, 3/207 Bouverie Street, Carlton, Melbourne, 3053, Australia; 6 School of Public Health, Sydney Medical School, University of Sydney, Sydney, NSW, 2006, Australia; 7 NHMRC Clinical Trials Centre, University of Sydney, Locked bag 77, Camperdown, Sydney, NSW, 1450, Australia; 8 Royal Women’s Hospital, Melbourne, Locked Bag 300, Grattan St & Flemington Rd, Parkville, Melbourne, VIC, 3052, Australia; 9 Present address: Department of Medical Epidemiology and Biostatistics, Karolinska Institutet, Nobels väg 12 A, P.O. Box 281, SE-171 77, Stockholm, Sweden; 10 Present address: School of Public Health and Community Medicine, University of NSW, Sydney, Australia

## Abstract

**Background:**

The National Cervical Screening Program in Australia currently recommends that women aged 18–69 years are screened with conventional cytology every 2 years. Publicly funded HPV vaccination was introduced in 2007, and partly as a consequence, a renewal of the screening program that includes a review of screening recommendations has recently been announced. This study aimed to provide a baseline for such a review by quantifying screening program resource utilisation and costs in 2010.

**Methods:**

A detailed model of current cervical screening practice in Australia was constructed and we used data from the Victorian Cervical Cytology Registry to model age-specific compliance with screening and follow-up. We applied model-derived rate estimates to the 2010 Australian female population to calculate costs and numbers of colposcopies, biopsies, treatments for precancer and cervical cancers in that year, assuming that the numbers of these procedures were not yet substantially impacted by vaccination.

**Results:**

The total cost of the screening program in 2010 (excluding administrative program overheads) was estimated to be A$194.8M. We estimated that a total of 1.7 million primary screening smears costing $96.7M were conducted, a further 188,900 smears costing $10.9M were conducted to follow-up low grade abnormalities, 70,900 colposcopy and 34,100 histological evaluations together costing $21.2M were conducted, and about 18,900 treatments for precancerous lesions were performed (including retreatments), associated with a cost of $45.5M for treatment and post-treatment follow-up. We also estimated that $20.5M was spent on work-up and treatment for approximately 761 women diagnosed with invasive cervical cancer. Overall, an estimated $23 was spent in 2010 for each adult woman in Australia on cervical screening program-related activities.

**Conclusions:**

Approximately half of the total cost of the screening program is spent on delivery of primary screening tests; but the introduction of HPV vaccination, new technologies, increasing the interval and changing the age range of screening is expected to have a substantial impact on this expenditure, as well as having some impact on follow-up and management costs. These estimates provide a benchmark for future assessment of the impact of changes to screening program recommendations to the costs of cervical screening in Australia.

## Background

The National Cervical Screening Program (NCSP) in Australia commenced in 1991 and is a joint program of the federal Australian Government and individual state and territory governments [1,2]. The NCSP recommends cervical screening every 2 years for sexually active women from age 18–20 years up to 69 years [3]. Women with abnormal cytology are followed-up with repeat cytology or referred for diagnosis using colposcopy and biopsy (if indicated), with the follow-up time for cytology or decision to refer depending on the severity of the abnormal cytology and a woman’s screening history and age [4]. Treatment is recommended for women diagnosed with histologically-confirmed high-grade cervical intraepithelial neoplasia (CIN 2/3) [4]. Post-treatment “test-of-cure” management is recommended for women previously treated for high-grade precancerous lesions, commencing 12 months after the treatment until the woman tests negative on both human papillomavirus (HPV) testing and cytology on two consecutive occasions [4]. In 2010, the age-standardised screening program participation rates over the previous 2 years, 3 years and 5 years among eligible women aged between 20 and 69 years were 57.4%, 70.2% and 83.3% [5]. Participation was highest among women aged between 30 and 64 years, but was lower in younger and older women within the target age range (18–69 years) [5]. Cervical cancer incidence and mortality rates in Australia have reduced by over 50% since the introduction of organised screening to 6.8 and 1.8 per 100,000 women, respectively in 2007 [5].

The National HPV Vaccination Program in Australia commenced in 2007. This comprises a school-based program, generally targeting girls in the first year of high-school (approximate age 12–13 years), and a catch-up program, running until the end of 2009, which provided vaccination for females aged between 13 and 26 years. Vaccination coverage in females aged between 18 to 26 years over the course of the catch-up program was between 30 and 38% for the full three-dose course [6]. In 2010, the oldest women in the catch-up cohort were aged 29 years. Many of the women in the catch-up cohort were expected to have participated in the cervical screening program.

Initial ecological data show an apparent early effect of vaccination in reducing high grade abnormalities in 2009–2010 in women less than 18 years, but not yet in older age groups [5,7]. There is expected to be a reduced risk of high grade abnormalities and invasive cervical cancer in young vaccinated women, and this has prompted consideration of increasing the age of starting screening. Even prior to the advent of vaccination, an increased screening interval and a later age of starting screening are supported by a large body of international evidence [8-10]. A process of Renewal (review) of the NCSP in Australia was announced in November 2011 [11]; this will involve considering new technologies, and reviewing the interval and age range of screening; if changes are implemented this would be expected to have a substantial impact on screening program costs. This study aimed to use a calibrated and validated model to provide a benchmark for future changes to the cervical screening program by quantifying screening program resource utilisation and costs in the year 2010.

## Methods

A deterministic Markov model, coupled with a dynamic model of HPV transmission, was constructed to simulate cervical screening in Australia. This single and multiple cohort model platform, adaptable to different settings, has been previously used to evaluate changes to the cervical screening interval in Australia and United Kingdom [12,13], to assess the role of alternative technologies for primary screening in Australia, New Zealand, and England [14,15], to assess the role of HPV triage testing for women with low-grade cytology in Australia and New Zealand [16],to assess the role of HPV testing in post-treatment management for women treated for CIN [15,17] and to evaluate the cost-effectiveness of alternative screening strategies and combined screening and vaccination approaches in rural China [18,19]. The dynamic model of HPV transmission is implemented in Microsoft Visual C++, and the Markov model is implemented in TreeAge Pro 2008 (Release 1.3.2, TreeAge Software, Inc., MA, USA), with a Microsoft Office Excel interface integrated with Visual Basic for multiple cohort implementations.

In this study, we modelled the natural history of HPV infection and CIN to simulate the progression and regression of women’s underlying health state of those infected with HPV. A woman’s screening outcome was modelled as the interaction between the test characteristics of the screening/diagnostic test (the positivity rate for each underlying health state) and the woman’s true underlying health state. Disease recurrence was modelled as women’s underlying health state progression from a no CIN state to CIN2+ after treatment at a specific annual rate. Recurrent CIN2+ could be detected during a visit, depending on the test characteristics of the screening/diagnostic test [14,16]. More details of model’s structure, assumptions and data source used are described in the following sections.

### Model of the natural history of HPV, precancer and cervical cancer

The natural history model simulates the development of cervical precancer and invasive cervical cancer in cohorts of women in Australia. The age-specific HPV incidence is obtained from a dynamic model of HPV transmission in Australia, [20,21] which simulates the natural history of HPV in females of all ages, using survey data on sexual behaviour in the community to predict HPV incidence by single year of age. The natural history model was adapted from a previously published model [12] and, after including screening and management (see below), was calibrated to Australian data on the age-specific prevalence of oncogenic HPV in cytologically-normal women, observed rates of histologically confirmed high-grade lesions, and cervical cancer age-specific incidence and mortality rates [13,14,16]. The age-standardised annual progression rate from CIN3 to asymptomatic localised cancer was calibrated to be 1.3%, consistent with the available data [22].

### Model of cervical screening, diagnosis and treatment

The details of the structure, assumptions, and the data source used in the Australia screening model have been previously described [13,14,16]. The screening component in the model was constructed according to current management guidelines for women with screen-detected cervical abnormalities in Australia [4]. The model contains detailed structures for modelling the management of cervical abnormalities. Briefly, women with a high-grade cytology result are referred for colpsocopic assessment; women with a low-grade cytology result are referred for further colposcopic assessment if they are found to have a recent (in the past 24 months) low-grade or worse cytology result or if the women is older than 30 years and has no history of negative cytology in the previous 2 years, otherwise, a 12 months follow-up with cytology test is recommended; women with negative cytology result are referred to have a follow-up cytology test at 12 months if they had a recent low-grade or worse cytology result, otherwise a 2-yearly routine screening is recommended [4]. Post-treatment management was modelled according to the recommendations of the relevant national guidelines [4]. The guidelines recommend that women treated for high-grade CIN are followed-up at 4–6 months after treatment with cytology and colposcopic examination, and then undergo annual follow-up with cytology and HPV testing, starting from 12 months after treatment, until they have tested negative by both cytology and HPV test on two consecutive occasions. The model takes into account the age distribution of starting screening and observed age-specific screening behaviour for women aged 18 years and older (including screening occurring in women older than the target age of screening); this was informed by analysis of data from the period 1997–2007 from 6.3 million satisfactory cytology tests from the Victorian Cervical Cytology Registry (VCCR). By directly modelling observed behaviour, the model captures the costs and effects due to both early re-screening and any late re-screening in different age groups.

The test characteristics for conventional cytology and HPV testing (currently used as a test-of-cure), were obtained from systematic reviews of the literature and were consistent with local observed data on cytology call rates [14]. Test characteristics for colposcopy were based on an analysis of the colposcopy-histology correlation from a database of approximately 21,000 colposcopies conducted in Victoria [14,16]. The failure rate of treatment for high grade CIN, and post-treatment recurrence rates were obtained from a meta-analysis of the international literature [14,16]. Stage-specific cervical cancer survival used in the model was derived from registry data from the state of New South Wales [14,16]. Age-specific deaths from causes other than cervical cancer were calculated using all-cause mortality after subtracting the cervical cancer mortality rate [23].

### Cost collation

Medicare, Australia’s universal health care system, reimburses procedures included on the Medicare Benefits Schedule (MBS). A health services perspective was taken in this study, considering only the costs to government related to screening, referral, diagnosis, treatment and follow-up of cervical abnormalities and the treatment of cervical cancer; overheads related to administration and promotion of the screening program, incentives to practitioners, and women’s out-of-pocket costs were not included.

Costs for screening, diagnostic and treatment procedures were derived with the aid of an expert advisory panel to the Medical Services Advisory Committee (MSAC) [14,16] based on current clinical practice and the item cost of the component services, which were obtained from MBS Online (2010) for outpatient medical services [24], National Hospital Cost Data Collection Round 13 (2008–09, public) for inpatient services [25] and the Pharmaceutical Benefits Schedule (PBS) Online (August 2008) [26] where applicable. The advisory panel comprised members/nominees from the Department of Health of the federal and state governments, the Royal Australian and New Zealand College of Obstetricians and Gynaecologists, the Royal College of Pathologists of Australia, the Royal Australian College of General Practitioners, the Australian Society of Cytology, and Consumers. The final methods and costs used were reviewed and approved by the panel.

We have previously described the methods for aggregating costs related to cervical screening in Australia in 2008 [14,16]. In the current study, we updated the item costs to 2010 and calculated aggregate costs using the same methods as previously described. Table 1 shows the summary of aggregate costs used by the model; full details of calculation of the aggregate costs are provided in the Additional file (See Additional file 1: Table S1-S10).

**Table 1:** **Aggregated cost data used in the model of cervical screening in Australia, 2010 (see Additional file****1****for associated cost items)**

**Cost item**	**Calculated aggregated cost in 2010**	**Itemised costs contained in the aggregated cost**	**Data sources**
***Cost of screening with cytology test***			
Having a cytology test alone (including $19.60 for cytology test)	$58.05	A weighted average cost of consultation, a cost of cytology test and a PEI	MBS online database 2010 [24] and Britt et. al. 2010 [27]
Having a cytology test after an unsatisfactory test	$67.23	An average cost of consultation, a cost of cytology test and a PEI	MBS online database 2010 [24]
***Cost of abnormal cytology follow-up***			
Abnormal cytology test result consultation and gynaecologist referral	$39.38	An average cost of consultation	MBS online database 2010 [24] and Britt et. al. 2010 [27]
Having a colposcopy without biopsy	$143.75	A cost of specialist consultation and a colposcopy	MBS online database 2010 [24]
Having a colposcopy with biopsy	$295.98	A cost of specialist consultation, a colposcopy, a biopsy and PEI	MBS online database 2010 [24]
Having a colposcopy without biopsy (with a cytology sample taken)	$171.60	A cost of specialist consultation, a colposcopy, a cytology test and a PEI	MBS online database 2010 [24]
Having a colposcopy with biopsy (with a cytology sample taken)	$315.58	A cost of specialist consultation, a colposcopy, a biopsy, a cytology and PEI	MBS online database 2010 [24]
***Cost of treating a precancerous lesions and follow-up women treated for CIN2/3***			
Treatment for precancerous lesions	$1,263.23	A weighted average cost of various high-grade lesions treatment procedures including ablation therapy, excision therapy and hysterectomy	MBS online database 2010 [24] and DRG [25]
Follow-up for women treated for CIN 2/3 at 4–6 months after treatment (using cytology test and colposcopy)	$130.65	A cost of specialist consultation, a cytology, a colposcopy and PEI	MBS online database 2010 [24]
Follow-up for women treated for CIN 2/3 at 12/24 months after treatment (using cytology test and HCII)	$131.23	A weighted average cost of consultation, a cytology, a HPV testing and PEI	MBS online database 2010 [24]
***Cost of work–up for cancer diagnosis***			
Localised cancer	$1,820.98	A weighted average cost of various cancer work-up diagnosis procedures including colposcopy, chest x-ray, CT scan, PET scan, MRI, bone scan and cystoscopy.	MBS online database 2010 [24], DRG [25] and expert opinions [14,16]
Regional cancer	$2,007.69		
Distant cancer	$1,979.24		
***Cost of cancer treatments***^†^			
Localised cancer treatment	$13,115.93	A weighted average cost of surgical (conisation, hysterectomy, radical hysterectomy and exenteration) and non surgical treatments (radiationtheraphy, adjuvant radiation therapy and chemo-radiation) received by cancer patients.	MBS online database 2010 [24], DRG [25], and expert opinions [14,16]
Regional cancer treatment	$32,048.22		
Distant cancer treatment	$24,250.30		
Terminal care	$24,250.30		Assumption

*PEI*, Patient episode initiation; *MBS*, Medical benefit schedule; *DRG*, diagnosed-related group; *CIN2/3*, cervical intraepithelial neoplasm grade 2 or 3; *HCII*, Hybrid Capture II.

^†^ The weighted average cost of cancer treatment was derived from the cost data by FIGO stage, which was grouped to broadly represent the extent of disease categories. The cost of cancer treatment varies significantly between FIGO 1a1 ($7,330.55), 1a2 ($13,164.32), 1b2 ($16,270.50) and the more advanced stages ($24,250.30-$32,458.30). See Additional file 1: Table S7–S10 for further details of the calculation of cancer treatment costs.

The aggregate cost of cytology comprised a medical consultation cost and a pathology cost. The consultation cost takes into account the range of practitioners who can collect cytology samples, and also that the purpose of consultation may be for multiple reasons. In the case where a second cytology test is performed after an unsatisfactory test, we assumed that the full cost of GP/specialist consultations was applicable. Abnormal cytology results were assumed to incur an additional consultation cost for conveying to the woman the cytology result. The model also assumed that a cytology test was performed during 85% of colposcopy visits, based on data from a large colposcopy database [14,16]. In Australia, currently the Medicare rebate for cytology test is the same regardless of whether or not the women comply with the recommended screening or follow-up interval.

The cost of treatment for precancerous lesions was composed of the weighted-average of the costs of ablational treatment, excisional treatment and hysterectomy. The weighting for each treatment type was informed by Medicare data on the number and type of treatments claimed in 2010, which was obtained from the MBS online database, [28] and an analysis of a large colposcopy database [14,16]. In the model, women treated for high-grade lesions attended for cytology and colposcopy within 6 months after treatment. The model assumes that in 6.4% of these women, initial treatment was unsuccessful and a second treatment was required, with a further follow-up visit 6 months later [14,16]. Women treated for high grade CIN lesions were assumed to be managed according to current guidelines by annual testing with cytology and HPV testing, starting from 12 months after treatment until the women tested negative for both tests on two consecutive occasions [4].

All cancer treatment costs, including the costs incurred in the year when cancer was diagnosed and the costs incurred over the subsequent years were applied as a one-off cost in the year of diagnosis, with the exception of terminal care costs which were applied in the year a patient died from cervical cancer. The distribution of cancer stage at diagnosis was based on data provided by the Queensland Gynaecological Cancer Centre and the Royal Women’s Hospital, Melbourne [14,16]. The data were provided according to International Federation of Gynecology and Obsterics (FIGO) stage and were then grouped to represent disease extent categories, consistent with the model’s structure. No national data were identified to inform the types and frequencies of procedures used for work-up and treatment for cervical cancer, therefore expert opinion was sought. The stage–specific work-up costs and cancer treatment costs were a weighted average of the relevant procedures, which were also based on expert opinion, as previously described [14,16].

### Model outcomes and comparison against observed data

The outputs of the model included age-specific predicted cost and rates of cytology test utilisation, colposcopies, biopsies, treatment for precancerous lesions, cancer incidence and cancer mortality. The model outputs for the annual number of cytology tests, numbers of histologically confirmed high grade lesions detected, and case numbers for incident cervical cancer and cervical cancer deaths have been previously validated by comparing against observed data [13,14,16,23,29]. For the current evaluation, we updated the population structure using the 2010 Australian female resident population [30]. We then compared updated predictions for the number of cytology tests against recent data [5]. Because cancer incidence and mortality data for the year 2010 were not yet available at the time of writing, population structures for the years 2004–2007 were applied for the purposes of comparing model outputs against observed age-specific cancer cases and deaths only, during which period the cervical cancer rates and mortality rates were relatively stable (at the time of writing, 2007 was the latest year where both the national cervical cancer incidence and death rates were publicly available).

We then used the model to estimate the expenditure and resource utilisation in the NCSP in 2010, including the cost and number of cervical screening tests, colposcopies, biopsies, treatments for precancerous lesions, post-treatment tests, cancer treatments, incident cervical cancer cases and cancer deaths. We also calculated the average per-woman costs related to cervical screening, diagnosis and treatment; firstly, per woman in the population and secondly, per woman participating in the screening program in that year, for adult women aged 18–84 years (in 2010, Australia had 8.5 million resident women aged between 18–84 years and more than 3.6 million women participated in NCSP over the 2 year period 2009-2010 [5,30]). The latter calculation takes into account that lower costs in some age groups may be due to lower coverage.

The model was also used to estimate expenditure, incident cancer cases and cancer deaths under the hypothetical scenario that no cervical screening had occurred. The estimated cost per incident cervical cancer case prevented and cost per cancer death prevented in 2010 were then calculated. The cost per life-year saved associated with current screening vs. no screening was also calculated. Costs and life-years were calculated from age 18 (the earliest age at which screening is recommended to start in Australia) with discounting at a rate of 5%. All costs are presented in 2010 Australian dollars ($A1 = $US0.85634 as at June 30, 2010).

### Sensitivity analysis

Sensitivity analyses were performed to assess the robustness of predicted expenditure to a subset of screening, cost and natural history parameter assumptions applied in the model. For the screening parameters, we examined the sensitivity of the model’s predictions to the assumptions around screening attendance and compliance with recommendations, the unsatisfactory rate of cytology and colposcopy, and test characteristics of cytology and colposcopy. For the aggregated unit cost parameters, alternate costs of treatment for precancerous lesions, cancer diagnosis and treatment and a lower 85% MBS reimbursement level were assessed. The sensitivity of the model’s prediction to a key natural history assumption was also assessed by halving and doubling the non-symptomatic invasive cancer progression probabilities.

### Ethical approval

Screening registry data, colposcopy data and other sources of data used in the model were de-identified, and the Cancer Council NSW Human Research Ethics Committee approved the transfer of these datasets to the researchers and their use in this modelled evaluation.

## Results

Model predictions for 2010 and recent observed data reported in Australia are summarised in Table 2. However, it should be noted that at the time of writing data for 2010 were not yet available for some screening program outcomes; therefore these predictions for 2010, which incorporate 2010 population information cannot be directly compared to the data for earlier years since the population structure and size changes over time.

**Table 2:** Model outcomes in Australia 2010, compared against latest observed data

	**Model predicted**	**Latest observed data**^†^
Number of cytology performed		
*20-69 years*	2 million	2 million
*All ages*	2.1 million	2.1 million
Number of low-grade cytology abnormalities detected in women 20–69 years	84,600	78,510^††^
Number of high-grade cytology abnormalities detected in women 20–69 years	27,700	28,491^††^
Number of women with abnormal cytology with a follow-up histological evaluation	33,200	38,859^††^
High-grade histology rate per 1,000 women screened	7.4	8.4
Age-standardised rate of cervical cancer incidence per 100,000 women^‡^		
*20-69 years*	8.9	9.3^‡‡^
*All ages*	6.6	7^‡‡^
Age-standardised rate of cervical cancer incidence per 100,000 women^‡^		
*20-69 years*	2.1	1.9^‡‡^
*All ages*	1.8	1.8^‡‡^

^†^ Data obtained from *Cervical Screening in Australia 2009–2010* report [5].

^††^ At the time of writing the latest available data was in 2009.

^‡^ Age-standardised to 2001 Australian population.

^‡‡^ At the time of writing the latest available data for cancer incidence was in 2008 and for cancer mortality was 2007.

### Overall costs and resource utilisation

The model estimated that the total expenditure for cervical screening program in 2010 was $194.8 million. Of this, the model predicted that 55% (~$107.6 million) was spent on cytology testing for routine cervical screening among women without a recent history of cervical abnormalities (within two years) and for follow-up of women with recent low-grade abnormalities. A further 11% (~$21.2 million) was spent on diagnostic procedures to follow-up abnormal cytology results, including colposcopies, cytology performed at colposcopies and biopsies; 23% (~$45.5 million) was spent on treatment for precancerous lesions and to provide post-treatment follow-up for “test-of-cure” management with cytology and HPV tests; and 11% (~$20.5 million) was spent on work-up and treatment for women diagnosed with invasive cervical cancer.

**Figure 1:**
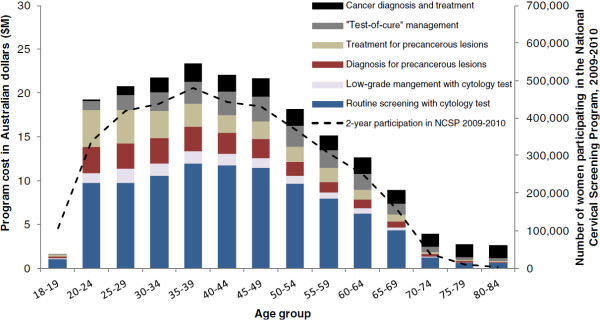
**Program cost by age and expenditure type, Australia 2010.** *Data obtained from *Cervical Screening in Australia 2009–2010*, published by Australia Institute of Health and Welfare (AIHW) in 2011 [5].

Figure 1 shows the estimated program expenditure by age and expenditure type, and women’s participation in NCSP over the 2-year period 2009–2010 (participation data obtained from the annual report published by the Australia Institute of Health and Welfare [5]). Screening with cytology comprises the major proportion (~50%) of expenditure in women aged less than 69 years. Diagnosis and treatment for precancerous lesions accounted for about one third of the expenditure in women aged less than 25 years (31-37%), but this proportion decreased gradually in older age groups. The cost of cancer diagnosis and treatment contributed less than 1% of the expenditure in women aged <25 years but the percentage increased in older age groups.

Of the $194.8 million total expenditure in 2010, about 95% (~$185.4 million) was estimated to have occurred in women aged 18 to 69 years. As shown in Figure 1, the highest expenditure was estimated to have occurred in women aged 35–39 years. Approximately half of the total expenditure (~$88.9 million) occurred between aged 30 and 49 years. This pattern of expenditure by age broadly mirrors patterns of participation by age (Figure 1).

**Figure 2:**
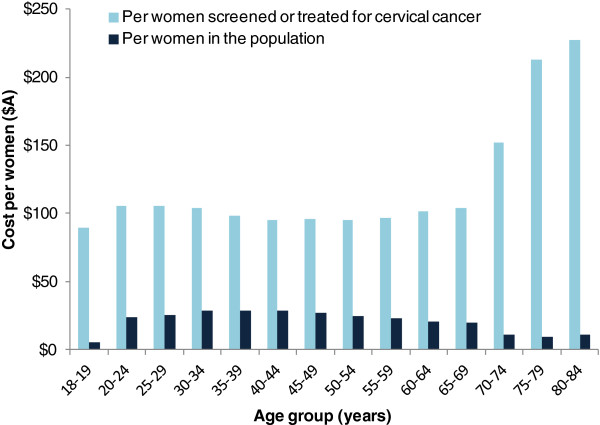
Age-specific average annual cervical screening-related expenditure per adult woman in the population and per adult woman screened, Australia 2010.

Figure 2 shows the estimated average screening cost in 2010 per woman by age. The estimated overall average screening cost per woman in the population aged between 18 to 84 years (including women not screened in the 2010 calendar year) was $23. As expected, the lowest average costs were in women outside the target age range for screening (<$11 in women aged <20 years and in women aged 70+ years), while the highest average cost ($27-29) was estimated to occur in women aged 30–49 years. When considering only women who participated in screening, and therefore taking into account coverage, the estimated average annual cost increased to $102 per adult woman. This varied from $89-$106 per woman screened or treated for cervical cancer for women aged between 18–69 years; and was greater than $150 per woman screened or treated for cervical cancer for women older than 70 years. Again, the difference in this average cost per woman screened or treated for cervical cancer among age groups is broadly attributable to the screening participation rate in women of different ages.

### Resource utilisation and costs associated with cytology

The model estimated that about 2.1 million cytology tests were performed in 2010 (including primary screening tests, repeat tests after an unsatisfactory result, tests collected during colposcopy and tests conducted for follow-up of women treated for precancerous lesion) at a cost of over $118 million in 2010 (Table 3). Of the 2.1 million cytology tests, more than 1.8 million (associated with a cost of ~$107.5 million) were performed as part of cervical screening (about 1.7 million cytology tests were performed among women without recent history of cervical abnormalities and approximately 188,900 tests were performed to follow-up women with recent low-grade abnormalities) and 160,600 cytology tests (associated with a cost of ~$10.8 million) were performed for test-of-cure management of women previously treated for histologically-confirmed high-grade cervical abnormalities. The remaining cytology tests were performed during colposcopy. About half of the overall cytology-related costs were incurred among women aged 30–49 years. The direct test cost was estimated to account for approximately one third of the total, with the remainder attributable to GP or specialist consultation fees and additional benefits paid to laboratories.

**Table 3:** Estimated annual number of cytology tests and associated costs (including consultation time and laboratory costs) by age group, Australia 2010

**Age group (years)**	**Cytology test conducted for primary routine screening**^†,††^		**Cytology test conducted for follow-up of low-grade abnormalities**^††,‡^		**Cytology test conducted as part of "test-of-cure" management after treatment for high-grade CIN**^††^		**Overall annual number of cytology test (No. of test conducted for routine screening or follow-up of low-grade abnormalities)**^‡‡^	**Annual cost related to cytology testing (cost related to cytology test conducted for routine screening or follow-up of low-grade abnormalities)**	***% total***
	**No. of tests**^‡‡^	**Related cost**	**No. of tests**^‡‡^	**Related cost**	**No. of tests**^‡‡^	**Related cost**			
**<20**	17,800	$1.0 M	600	$32,900	60	$3,900	19,100 (18,400)	$1.1 M ($1.1 M)	*1%*
**20-24**	167,100	$9.7 M	20,000	$1.2 M	4,100	$0.3 M	201,600 (187,000)	$11.2 M ($10.9 M)	*9%*
**25-29**	166,900	$9.7 M	28,700	$1.7 M	10,500	$0.7 M	215,900 (195,600)	$12.1 M ($11.4 M)	*10%*
**30-49**	784,200	$45.7 M	91,300	$5.2 M	76,300	$5.1 M	981,800 (875,400)	$56.0 M ($50.9 M)	*47%*
**50-69**	482,700	$28.1 M	44,500	$2.6 M	61,200	$4.1 M	600,000 (527,200)	$34.8 M ($30.7 M)	*29%*
70+^*^	41,300	$2.4 M	3,900	$0.2 M	8,300	$0.6 M	54,800 (45,200)	$3.2 M ($2.6 M)	*3%*
**20-69**	1,600,900	$93.2 M	184,400	$10.6 M	152,200	$10.2 M	1,999,200 (1,785,300)	$114.1 M ($103.9 M)	*96%*
**0-84**	1,660,000	$96.7 M	188,900	$10.9 M	160,600	$10.8 M	2,073,100 (1,848,900)	$118.3 M ($107.5 M)	*100%*

^†^ When attending for primary screening for women without abnormal results from the previous two or more consecutive cytology tests.

^††^ Repeat smears conducted during colposcopy examination, precancerous lesion treatments and post-treatment follow-up are not included.

^‡^ Follow-up of cytological low grade abnormality, or of histologically-verified CIN1 or less; or of women referred with a cytological high-grade but with no abnormality verified at colposcopy/biopsy.

^‡‡^ Numbers less than 100 are rounded to the nearest 10. Numbers greater than 100 are rounded to the nearest 100.

^*^ Up to 84 years.

The model also estimated 87,500 and 28,600 cytology tests were found to have low-grade and high-grade abnormalities respectively in women of all ages in 2010; 84,600 of these low-grade abnormalities and 27,700 of these high-grade abnormalities were found in women aged 20–69 years.

### Resource utilisation and costs associated with diagnostic evaluations

The model predicted that about 70,900 colposcopy and 34,100 histology evaluations were performed in 2010 (not including multiple biopsies taken at the same colposcopy), costing $21.2 million in 2010 (Table 4). This includes the cost of specialist visits, colposcopy tests and associated cytology tests, biopsy tests and other histopathology-related expenditure. About half of the costs were incurred in women aged 30–49 years.

**Table 4:** Estimated annual number of diagnostic evaluations and treatment for precancerous lesions and related costs by age group, Australia 2010

**Age group (years)**	**Diagnostic procedures for precancerous lesions**				**Treatment for precancerous lesions and post-treatment follow-up**			**Colposcopy to treatment ratio**
	**No. of colposcopy evaluations**^†,††^	**No. of histological evaluations**^†,††^	**Related cost**	**% total**	**No. of precancerous lesion treatments**^**†,‡**^	**Related cost**	**% total**	
**18-20**	700	400	$0.2 M	1%	200	$0.3 M	1%	3.2
**20-24**	10,900	5,900	$3.0 M	14%	3,300	$5.2 M	11%	3.3
**25-29**	10,500	5,700	$2.9 M	14%	3,000	$5.5 M	12%	3.5
**30-49**	33,900	16,900	$10.2 M	48%	7,800	$20.1 M	44%	4.3
**50-69**	13,500	4,800	$4.4 M	21%	4,100	$12.8 M	28%	3.3
**70+**^‡‡^	1,400	500	$0.5 M	2%	400	$1.6 M	3%	3.1
**20-69**	68,800	33,200	$20.5 M	97%	18,200	$43.6 M	96%	3.8
**0-84**	70,900	34,100	$21.2 M	100%	18,900	$45.5 M	100%	3.8

^†^ Rounded to the nearest 100.

^††^Multiple biopsies taken at the same colposcopy were not included.

^‡^ Includes procedures performed in women without prior confirmation of high-grade disease (e.g. women with unsatisfactory colposcopy findings) who may have been found to have a low-grade histological outcome.

^‡‡^ Up to 84 years.

### Resource utilisation and costs associated with treatment for precancerous lesions

The model estimated that about 18,900 treatments for precancerous lesions were performed in 2010, and the associated costs of treatment and post-treatment follow-up were $45.5 million (Table 4). This includes the cost of specialist consultation, treatment procedures, and other related procedures (such as the colposcopy visit where treatment occurs and histopathology), as well as test-of-cure management. The model predicted that about 44% of the precancerous treatments and associated follow-up cost were incurred among women aged 30–49 years. We also estimated the colposcopy to precancer treatment ratio, which gives an indication of the overall efficiency of the screening process in referring women requiring treatment, where a value closer to one indicates more efficient referral (Table 4). This was estimated to be 3.8 overall; the highest colposcopy-to-treatment ratio (4.3) was in women aged 30–49 years and the lowest ratio (3.5 or less) was in women younger than 29 years or older than 50 years; the lower ratio in women under the age of 30 years is assumed to be due, in part, to the recommendation to repeat annual smears in women with low grade cytology but without a history of cervical abnormalities in the last 2 years before referral to colposcopy [4,5]; this is predicted to result, in this age group, in women who are referred to colposcopy being more likely to harbour confirmed high grade disease.

### Resource utilisation and costs associated with cancer treatment

Table 5 shows, firstly, a comparison of model predictions for cervical cancer cases and deaths over the period 2004–2007, with observed average case numbers over the period. Table 5 also shows the predicted number of cases and deaths after applying the 2010 population structure. The model estimated that 761 cases and 213 deaths occurred in 2010 overall. The estimated associated cost of cervical cancer diagnosis and treatment was $20.5 million, with 94% of diagnosis and treatment related costs incurred in women aged over 30 years.

**Table 5:** Estimated annual number of cancer treatments and related costs by age group, Australia 2010

**Age group (years)**	**Cervical cancer cases**			**Cervical cancer deaths**			**Related cost in 2010**	**% total**
	**Model predicted**^†^		***Observed (average 2004–2007)***	**Model predicted**^†^		***Observed (average 2004–2007)***		
	**in 2010**	**Average in 2004-2007**		**in 2010**	**Average in 2004-2007**			
**<20**	0	0	*1*	0	0	*0*	$0.0 M	0%
**20-24**	16	15	*11*	0	0	*1*	$0.3 M	1%
**25-29**	46	39	*41*	5	4	*3*	$0.9 M	5%
**30-49**	341	328	*338*	70	67	*50*	$7.7 M	37%
**50-69**	235	206	*208*	79	69	*76*	$7.0 M	34%
**70+**^††^	121	115	*102*	59	57	*57*	$4.6 M	23%
**20-69**	639	588	*598*	154	140	*130*	$15.8 M	77%
**0-84**	761	703	*701*	213	197	*187*	$20.5 M	100%

^†^ The numbers are rounded.

^††^ Up to 84 years.

### Cost per cervical cancer case and death prevented

The model predicted that 2,475 incident cancer cases and 894 cancer deaths at an associated cost of $75 million (for cancer treatment related costs) would have occurred in 2010 in the absence of cervical screening. Therefore, the number of cases prevented by the current organised program is estimated to be 1,714 (69% reduction) and the number of deaths prevented is estimated to be 681 (76% reduction). It is possible to calculate estimates for cost per cancer and death prevented, as well as the cost-effectiveness of the screening program overall; but these estimates must be interpreted with caution for several reasons. Firstly, such estimates assume no change to risk experienced in different age cohorts, which due to changing patterns of sexual behaviour may not be the case. Secondly, costs to prevent cancer and deaths occurring in 2010 were mainly incurred in past years, due to the inherent delay in cervical cancer development. Thirdly, the estimated costs of cancer treatment in the hypothetical counterfactual scenario of no screening do not take account of further centralisation of treatment facilities if the burden of disease was higher. However, bearing in mind these limitations, the estimated cost per case prevented was $69,400, and the estimated cost per death prevented was $174,600 in 2010, and the estimated incremental cost-effectiveness ratio of the organised screening program, compared to no screening, was $33,000 per life-year saved.

### Sensitivity analysis

**Figure 3:**
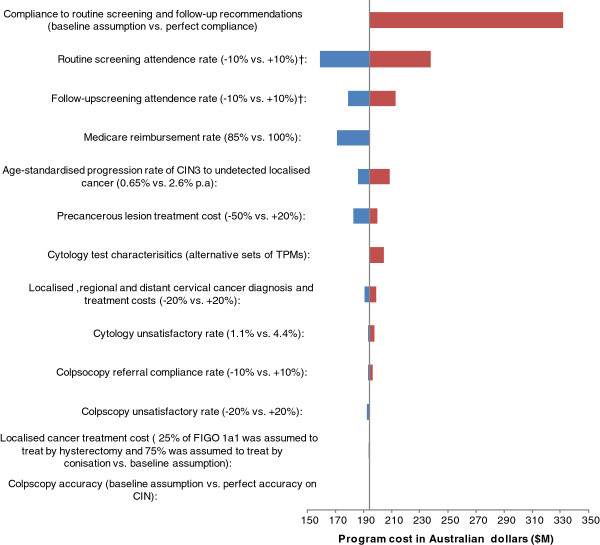
**Sensitivity analysis for total program expenditure, Australia 2010.** † Cumulative attendance at each interval since last screened to 10 years were varied.

Figure 3 summarise the findings of sensitivity analysis on the predicted total program expenditure. The model prediction is found to be most sensitive to assumptions around screening behaviour. Under the assumption that all women who participate in the screening program comply perfectly to the 2-yearly routine screening and 12 months follow-up recommendations, the estimated program cost increased to ~$340 million. This is 70% higher than the base case estimate which modelled the observed screening behaviour in the population. The estimated program cost was also somewhat sensitive to the assumptions about the level of Medicare reimbursement, CIN natural history and precancerous lesion treatment costs, and was found to be moderately sensitive to assumptions about test characteristics and the unsatisfactory rate of cytology and colposcopy, cost of cancer diagnosis and treatment and the compliance with colposcopy referral.

## Discussion

We have constructed a comprehensive natural history and cervical screening model, using observed data on actual screening behaviour, and extensively calibrated and cross-validated outputs against observed data from the NCSP in Australia. We estimate that the program cost to government, excluding administrative overheads, was approximately $194.8 million in 2010. In total, about $174.2 million (89%) of the total expenditure was estimated to be associated with the prevention of invasive cervical cancer (primary screening of asymptomatic women, follow-up of women with cervical abnormalities, and diagnosis and treatment for women with precancerous lesions). Within this, a total of 1.7 million primary screening smears costing $96.7 million were estimated to have been conducted, a further 188,900 smears costing $10.9 million were conducted to follow-up low grade abnormalities, 70,900 colposcopy and 34,100 histological evaluations costing $21.2 million were performed, and about 18,900 treatments for precancerous lesions were performed, costing $45.5 million after accounting for both the treatment and post-treatment follow-up. We also estimated that $20.5 million (11%) of the total expenditure was associated with work-up and treatment for the approximately 761 women who were diagnosed with invasive cervical cancer, and care for the 213 women who died from cervical cancer.

To our knowledge this is the first study that provides such detailed estimates of resource utilisation in relation to any organised cervical screening program in a developed country. The findings of this evaluation provide a comprehensive source of information on the NCSP which has not previously been available. Screening histories for women who participate in the cervical screening program are collected by State and Territory level Pap test registers [1,2] and the collected data are collated by the Australia Institute of Health and Welfare (AIHW) to produce annual public reports summarising participation rates in the program, early re-screening rates, abnormalities detected by cytology and histology, cervical cancer incidence and cervical cancer mortality in Australia. The most recent report for the period 2009–2010 contains expanded information on performance indicators and also includes overall numbers of cytology and histology tests [5]. However, information on the detailed breakdown of the indications for cytology testing (primary screening, follow-up, or test-of-cure), and the resource utilisation associated with colposcopy, biopsy, treatment, and test-of-cure is either not yet uniformly collected (as for in the case of colposcopy) or has not yet been able to be routinely reported. Where comparison was possible, we found good agreement between our findings and the data published by the AIHW. We estimate that a total of about 2.1 million smears were performed in 2010, 2 million of these were performed in women aged 20–69 years; which is consistent with the number of tests performed in 2010 recently published by AIHW (2,109,131 in women of all ages and 2,025,860 in women aged 20–69 years) [5]. We estimate that there were 84,600 and 27,700 cytology tests which were found to have low-grade and high-grade cervical abnormalities respectively in women aged 20–69 years, and that 33,200 women with abnormal cytology had a follow-up histological evaluation. These findings are broadly consistent with the number of cytology tests with an abnormal result in 2010 (78,510 tests associated with low-grade abnormalities and 28,491 tests associated with high-grade abnormalities), and the number of cytology tests performed which were followed by a histological evaluation test in 2009 (38,859 tests) published by AIHW [5].

The previously available information on the cost of cervical cancer screening in Australia is limited. A 1993 report of the annual cost of the National Cervical Screening Program to government estimated these costs as $138 million; [31,32] applying the health services consumer price index [33,34] this is equivalent to $302 million in 2010, but it is not clear whether downstream diagnostic and treatment costs (or overheads costs) were taken into account in the estimate. Annual reports on Australia’s national public health expenditure published by the AIHW have included some information about expenditure on the cervical screening program in 2007, the AIHW reported that cervical screening-related expenditure by federal and state governments was $113.2 million, with an average expenditure of $5.33 per person (including both males and females) [35]. Of the $113.2 million, 70% ($79.3 million) was spent by the federal government on screening-associated costs including incentive payments, Medicare benefits for general practitioner consultations, pathology testing and other benefits related to collecting cytology samples, and departmental expenses [35,36]. The remaining $33.9 million was spent by State and Territory governments on program implementation and promoting community awareness of the screening program [35]. Although the overall estimate includes some of the direct costs included in our calculations, and additionally includes overheads and program promotion costs not included in our estimates, it does not appear to include costs associated with follow-up and diagnostic management or treatment for precancerous lesions. Therefore the findings are not directly comparable with our results. In 2005–06, the AIHW provided an estimate of government expenditure for women presenting with symptoms indicative of cancer of $20.1 million, [36] which appears broadly comparable with our estimated costs for cancer work-up and treatment of $21.5 million.

The total expenditure on health goods and services in Australia in FY2009-10 has been estimated at $121.4 billion by the AIHW, [37] and therefore our estimate suggests that secondary prevention with cervical screening comprises approximately 0.16% of the health-related expenditure in Australia. However, our findings represent an underestimate of the total costs related to cervical cancer prevention and treatment, for several reasons. Firstly, the total costs also include costs related to primary prevention via the implementation of the National HPV Vaccination Program, which was beyond the scope of the current evaluation. Secondly, we did not consider administrative overheads for the screening program, which may include substantial costs related to implementation at the State and Territory level as well as practice incentive payments for screening in women aged 20–69 years who have not had a cervical smear in the last four years, and other incentives for practices that engage with the program and screening registers [38]. In this study we did not incorporate overhead costs related to running the screening registries and sending screening reminder letters. These costs vary from state to state and are not available in the public domain. They are not readily amenable to the type of modelled analysis used in our paper since the overhead costs are not related to the number of women screened, but are likely to relate to state-specific infrastructure and funding arrangements. The NCSP Renewal process includes a process to explore an option for a real or virtual national register system [11] and also an option to change from the current reminder-based system to an invitational system of call and recall. In consequence the administrative overheads for the screening program may potentially change. Additionally, because our evaluation was conducted from the health-services perspective, we considered only the costs to government. We did not consider additional societal costs to women or their families that may be related to cervical screening. For example, in the state of NSW, up to 30% of women choose to have an adjunctive liquid-based cytology smear, for which they pay out of their own pocket [14,39], and therefore these costs are incurred outside the screening program. If a societal perspective was taken, this cost as well as women’s out-of-pocket expenses for transportation, lost of productivity, capital cost and depreciation, and other program operational costs including regular program overheads, screening participation incentives, and the costs associated with awareness and other health promotion campaigns would need to be included in the evaluation.

Although our findings appear to be in good agreement with the available data, the predicted levels of resource utilisation depend on a number of factors. We assessed screening behaviour in women of various age groups and with different screening histories over a 10 year period using data from Victoria (1995–2007), and then projected forward to estimate behaviour and the consequent resource utilisation in the year 2010. Newer processes for management of low grade abnormalities, and post-treatment management and test-of-cure only began to be rolled out from mid-2006, [4] and therefore information on compliance with these recommendations is much more limited than is the case for attendance for routine screening. While we were able to derive compliance estimates based on the data from the VCCR after the introduction of the updated guidelines, practices and behaviour may have changed over time. Any differences between the assumed and actual behaviour will affect the accuracy of our estimates of resource utilisation in the program. Additionally, based on data showing that cervical cancer incidence and mortality rates have stabilised since about 2003–2004, [40] we assumed that rates in 2010, which were not yet reported at the time of writing, would be similar to those observed over the period 2004–2007; again, if actual rates are eventually found to be substantially different from these levels this would have an impact on the interpretation of our findings. For the current analysis we also assumed that the population-based HPV vaccination program, rolled out in 2007, did not have a discernable impact on the rate of detected precancerous abnormalities in adult women (aged 18 years or older) within the screening program by 2010. This assumption is supported by the findings of a recent study in Victoria, which identified a reduction in the incidence of histologically-confirmed high-grade precancerous lesions in females younger than 18 years by the end of 2009 (which may be an early marker of the effects of the vaccination program), but identified no significant effect on the incidence of high grade abnormalities in women above the age of 18 years at this stage [7].

As a strength of our analysis, we used extensive screening data from VCCR to explicitly model for the heterogeneity of re-screening rates based on measured covariates for age, time elapsed since last screen and recommended follow-up after last screen. We did not model for residual individual-level heterogeneity in the re-screening rates due to unmeasured covariates, due to a lack of information on such covariates to the required level of detail. Given the explicit modelling of covariates for re-screening rates, we would expect that residual individual-level heterogeneity to have limited bias on the measures of cost-effectiveness.

The annual expenditure on cervical screening and treatment for CIN (excluding cancer treatment-related expenditure) in Italy, France and UK has previously been estimated to be €181.5 million (~A$232 million) with an estimated 6.4 million women screened in 2005 [41], €196.5 million (~$251 million) with 6.1 million women screened in 2004 [42] and £157 million (~$249 million) with 3.4 million women screened in 2006–07 [43], respectively. Our estimates, based on these data, of the associated estimated annual expenditure on cervical screening and treatment for CIN per women screened in Italy (~$36), France (~$41) and UK (~$73) is lower than the estimated ~$91 per woman screened in Australia (this estimate excludes a calculated $11 per women in Australia for cancer treatment, for comparability with the other estimates). The differences might be due to a number of factors, and it is not clear in how much detail the costs in the other evaluations have been modelled. Some of the factors impacting the comparison, might include, for example, the differences in screening recommendations (e.g. screening interval and management of abnormal cytology), screening participation, local screening and diagnostic test performances, the underlying disease prevalence in the population and health system costs.

We have estimated that the average annual cost of screening per adult woman in the population is $23, and that a substantial proportion of this is related to delivery of primary screening tests and follow-up of low grade cytological abnormalities. In the future, due to the effects of the National HPV Vaccination Program, it is expected that the number of cervical abnormalities and cases of invasive cervical cancer detected by the organised cervical screening program will decrease as more vaccinated women enter and age within the screening program over time. An ongoing body of work will use the model platform described here to predict the magnitude and timing of the impact of the National HPV Vaccination Program on resource utilisation in young women within the screening program, and will also assess the impact of changing screening recommendations on resource utilisation, costs, distribution of cancer stage at diagnosis and the effectiveness of screening. For example, we have previously used the platform to evaluate the impact of moving to a three-yearly screening interval under different systems of screening organisation (reminder-based versus call-and-recall organisation) [13], finding that three-yearly screening would be cost saving and would not have a substantial impact on the number of cervical cancer cases and cancer deaths. Ongoing work will also consider the impact of raising the age of starting screening, and the effect of further increases in screening interval which could be considered in conjunction with the implementation of primary HPV screening [44]. These evaluations, conducted using the calibrated and validated model platform described in the current study, are intended to assist policy-making in designing optimal strategies for cervical cancer prevention in future.

Some changes to screening programs may affect cancer incidence, but not, to the same degree, mortality because actions to increase compliance to recommendations may impact on stage distribution of detected cancers and thus mortality. Therefore, not only changes in cancer incidence but also changes in stage distribution in relation to screening and vaccination at different ages should be evaluated. The current model is calibrated to observed data on stage at diagnosis, and incorporates heterogeneity in screening uptake and compliance with screening and follow-up recommendations. These aspects of the model structure will allow some insight in future evaluations into how changes would affect not only numbers of cancer cases, but also age distribution, stage at diagnosis, and therefore survival, in women who are unscreened or underscreened, as well as those who attend regularly or are overscreened.

## Conclusions

We estimate that the total expenditure for the NCSP in Australia in 2010 was $194.8 million (excluding administrative overheads), and that the corresponding average annual cost of screening each adult woman in the population was $23. Approximately half of the total cost of the screening program was spent on primary screening with cytology; but the introduction of HPV vaccination, new technologies, increasing the interval and changing the age range of screening are expected to have a substantial impact on this expenditure, as well as having an impact on follow-up and management costs. This evaluation provides a benchmark for future assessment of the impact of HPV vaccination and of potential changes to screening program recommendations on the resource utilisation and costs of cervical screening in Australia.

## Abbreviations

AIHW: Australia Institute of Health and Welfare; CIN 2/3: Histologically-confirmed high-grade cervical intraepithelial neoplasia; DRG: Diagnosis related group; FIGO: International Federation of Gynecology and Obsterics; HPV: Human papillomavirus; MBS: Medical Benefits Schedule; NCSP: National Cervical Screening Program; PEI: Patient episode initiation; PBS: Pharmaceutical Benefits Schedule; VCCR: Victorian Cervical Cytology Registry.

## Competing interests

KC, KH, DG, SL and JT declare that they are involved as investigators in a new trial of primary HPV screening in Australia, which will involve support from the manufacturers of new test technologies. Other authors declared no conflict of interest. This study was funded by the National Health and Medical Research Council Australia (NHMRC Project Grant #1007518) and by Cancer Council NSW.

## Authors’ contributions

JBL led the development of the Australian screening model, performed the modelling analysis for this evaluation, participated in the analysis of model outcomes, prepared the tables and figures and participated in drafting the manuscript. KH prepared cost information for the model and guided the interpretation of cost outcomes. DG provided screening registry data and guided interpretation of these data. MS participated in model development, data analysis, manuscript drafting and was responsible for the collation and integration of epidemiological data into the model. MC led the analysis of screening registry data and conceived the basic design of the screening model. CN participated in model development. JF participated in manuscript drafting. SD and SL performed systematic review of cytology accuracy for the model and assisted in the specification of model screening and management pathways. PC participated in the model development for and the analysis of screening registry data and test probabilities matrix to inform cytology accuracy. YJK performed review of precancerous lesion treatment success rates and post-treatment recurrence rates. JT provided colposcopy data from a large database collected at Royal Women’s Hospital, Melbourne, and guided interpretation of these data. KC conceived and led the project, participated in all aspects of the analysis, and drafted the manuscript. All authors read and approved the final manuscript.

## Authors’ information

Jie Bin Lew BSc (Bioinformatics) MPH, Senior Research Programmer, Cancer Research Division, Cancer Council New South Wales, Sydney, Australia.

Kirsten Howard PhD, Senior Lecturer, School of Public Health, The University of Sydney, Sydney, Australia.

Dorota Gertig MBBS (Hons) MHSc ScD FAFPHM, Epidemiologist VCS/Medical Director VCCR, Victorian Cytology Service.

Megan Smith BE MPH, Cervical Modelling Program Manager, Cancer Research Division, Cancer Council New South Wales, Sydney, Australia.

Mark Clements PhD, Research Fellow, National Centre for Epidemiology and Population Health, Australian National University, Canberra, Australia. (Current position and address: Associate Professor, Department of Medical Epidemiology and Biostatistics, Karolinska Institutet, Stockholm, Sweden)

Carolyn Nickson PhD, Research Fellow, Centre for Women’s Health, Gender and Society, University of Melbourne, Victoria, Australia

Ju-Fang Shi PhD, Research Fellow, Cancer Research Division, Cancer Council New South Wales, Sydney & School of Public Health, Sydney Medical School, The University of Sydney, Sydney, Australia

Suzanne Dyer PhD, Systematic Reviews and Health Care Assessment, NHMRC Clinical Trials Centre, The University of Sydney, Sydney, Australia.

Sarah Lord MBBS MS (Epi), Epidemiologist, NHMRC Clinical Trials Centre & The Screening and Test Evaluation Program, The University of Sydney, Sydney, Australia.

Prudence Creighton BSc, Research Programmer, Cancer Research Division, Cancer Council New South Wales, Sydney, Australia. (Current address: School of Public Health and Community Medicine, University of NSW, Sydney, Australia)

Yoon Jung Kang BA MPH (Hons). PhD candidate, Cancer Research Division, Cancer Council New South Wales, Sydney, Australia

Jeffrey Tan MBBS MRCOG FRANZCOG, Consultant Gynaecologist, Royal Women's Hospital, Melbourne, Victoria, Australia

Karen Canfell DPhil, Sydney Rotary Research Fellow, Cancer Research Division, Cancer Council New South Wales & School of Public Health, Sydney Medical School, The University of Sydney, Sydney, Australia.

## Pre-publication history

The pre-publication history for this paper can be accessed here:

http://www.biomedcentral.com/1472-6963/12/446/prepub

## Supplementary Material

Additional file 1 **Table S1.** Average costs of medical consultation. **Table S2.** Problems managed per GP encounter – single service weighting. **Table S3.** Cost of screening test. **Table S4.** Cost of diagnostic procedures. **Table S5.** Cost of precancerous lesion treatment. **Table S6.** Cost post-treatment follow-up after CIN2/3 treatment. **Table S7.** Summary work-up and treatment by FIGO stage and disease extension. **Table S8.** Summary stage-specific cervical cancer work-up and treatment costs by FIGO stage. **Table S9.** Cost of surgical managements for cervical cancer treatment. **Table S10.** Cost of non-surgical managements. Click here for file
