# Supplementary material for: Expenditure and resource utilisation for cervical screening in Australia
Source: BMC Health Serv Res. 2012 Dec 5;12:446. doi: 10.1186/1472-6963-12-446 (PMC3548768; doi:10.1186/1472-6963-12-446)
Supplement: Additional file 1 — Table S1. Average costs of medical consultation. Table S2. Problems managed per GP encounter – single service weighting. Table S3. Cost of screening test. Table S4. Cost of diagnostic procedures. Table S5. Cost of precancerous lesion treatment. Table S6. Cost post-treatment follow-up after CIN2/3 treatment. Table S7. Summary work-up and treatment by FIGO stage and disease extension. Table S8. Summary stage-specific cervical cancer work-up and treatment costs by FIGO stage. Table S9. Cost of surgical managements for cervical cancer treatment. Table S10. Cost of non-surgical managements. [file 1472-6963-12-446-S1.docx]

Supplementary Material accompanying the article:

# Expenditure and resource utilisation for cervical screening in Australia

Jie-Bin Lew,^1^ Kirsten Howard,^2^ Dorota Gertig,^3^ Megan Smith,^1^ Mark Clements,^4,*^ Carolyn Nickson,^1,5^ Ju-Fang Shi,^1,6^ Suzanne Dyer,^7^ Sarah Lord,^2,7^ Prudence Creighton,^1,δ^ Yoon-Jung Kang,^1^ Jeffrey Tan^8^ and Karen Canfell^1,6§^

^1^ Cancer Research Division, Cancer Council NSW, 153 Dowling Street, Woolloomooloo, NSW 2011, Australia.

^2^ Screening and Test Evaluation Program, School of Public Health, University of Sydney, NSW 2006, Australia.

^3^ Victorian Cytology Service, 752 Swanston Street, Carlton VIC 3053, Australia.

^4^ National Centre for Epidemiology and Population Health, Australian National University, Canberra, ACT 2000, Australia.

^5^ Centre for Women’s Health, Gender and Society, University of Melbourne, 3/207 Bouverie Street, Carlton 3053, Australia.

^6^ School of Public Health, Sydney Medical School, University of Sydney, NSW 2006, Australia.

^7^ NHMRC Clinical Trials Centre, University of Sydney, Locked bag 77, Camperdown NSW 1450, Australia.

^8^ Royal Women's Hospital, Melbourne, Locked Bag 300, Grattan St & Flemington Rd, Parkville VIC 3052, Australia.

^*^ Present address: Department of Medical Epidemiology and Biostatistics, Karolinska Institutet, Nobels väg 12 A, P.O. Box 281, SE-171 77 Stockholm, Sweden.

^δ^ Present address: School of Public Health and Community Medicine, University of NSW, Sydney, Australia.

^§^ Corresponding author.

# Model assumptions for costs associated with cervical screening in Australia: Note on methods

The costs included in this modelled economic evaluation consisted of the costs related to screening, diagnosis, treatment and follow-up of cervical abnormalities, and to the treatment of cervical cancer, based on current Australian clinical practice. The unit costs for procedures were sourced from Medical Benefit Schedule (MBS) Online (March 2010)[1] for outpatient medical services, National Cost Data Collection (NCDC) Round 13 (2008-09, public)[2] for inpatient services and Pharmaceutical Benefits Schedule (PBS) Online [3], where applicable. Data on the usage of the resource item were based on PBS and MBS use statistics for 2010, obtained from Medicare Australia [4]. Literature sources online and advisory panel expert opinions were used, as necessary, to supplement data on local clinical practice.

## Cost of cytology sample collection and patient visits

The cost of screening with cytology includes a weighted-average cost of a medical consultation, a MBS fee for reading of a cytology test, and a patient episode initiation (PEI) fee. The cost used for medical consultation was $39.38 (see Supplementary Table 1). This cost consists of the weighted-average cost of medical surgery consultation in 2010 based on the number of each MBS item claimed in 2010, obtained from Medicare online database (URL: <https://www.medicareaustralia.gov.au/statistics/mbs_item.shtml>. Accessed on 1^st^ of May 2012). An additional weighting factor of 0.767 was applied to reflect that having a cytology test may not be the only reason for a consultation (see Supplementary Table 2). This weighted factor was derived from patient reasons for encounters published in the General Practice Activity data for 2009-10 by Australia Institute of Health and Welfare (AIHW)[5].

The model assumed 2.2% [6,7] of cytology tests would have a unsatisfactory result which required women to have a second cytology test. In the case of second cytology test after an unsatisfactory test, having a cytology test was assumed to be the only reason for the women to have a medical consultation and hence, the single-service adjustment factor was not used. Therefore, we assumed the cost of Pap test for the purpose of initial screen is $58.05 and $67.23 for the purpose of repeat cytology after the unsatisfactory test (see Supplementary Table 3).

**Supplementary Table 1. Average costs of medical consultation.**

| **Components** | **MBS item** | **Unit cost** | **Proportion of consultations** | **Weighted cost (Schedule Fee)** |
| --- | --- | --- | --- | --- |
| GP consultation - limited management | 3 | $16.00 | 2.84% | $0.46 |
| GP consultation, < 20 minutes | 23 | $34.90 | 75.09% | $26.21 |
| GP consultation, 20-40 minutes | 36 | $67.65 | 8.53% | $5.77 |
| GP consultation, > 40 minutes | 44 | $99.55 | 0.70% | $0.69 |
| Non-GP consultation, < 5 minutes | 52 | $11.00 | 0.07% | $0.01 |
| Non-GP consultation, 5-25 minutes | 53 | $21.00 | 3.16% | $0.66 |
| Non-GP consultation, > 45 minutes | 54 | $38.00 | 0.46% | $0.17 |
| Non-GP consultation, 25-45 minutes | 57 | $61.00 | 0.11% | $0.07 |
| Specialist consultation, initial | 104 | $82.30 | 3.91% | $3.22 |
| Specialist consultation, subsequent | 105 | $41.35 | 5.13% | $2.12 |
| **Weighted average cost per consultation** | |  | **100.00%** | **$39.38** |

*GP: General practitioner*

**Supplementary Table 2. Problems managed per GP encounter – single service weighting.**

| **Number of RFEs at encounter** | **Number of encounters^a^** | **Per cent of encounters^a^** | **Single service weighting** | **Weighted-average single service adjustment** |
| --- | --- | --- | --- | --- |
| One RFE | 58439 | 57.7% | 1 | 0.577 |
| Two RFEs | 30099 | 29.7% | 0.5 | 0.148 |
| Three RFEs | 12811 | 12.6% | 0.33 | 0.042 |
| **Total** |  | **100%** |  | **0.767** |

*^a^ Data are obtained from Britt et. al. (2010) – General practice activity in Australia 2009-10 (Table 6.2)[5]*

*REF: Reason For Encounter*

**Supplementary Table 3. Cost of screening test**

| **Components** | **MBS item** | **Unit cost (100% Schedule)** | **Weighting for single service** | **Cost (Initial screen)** | **Cost (repeat cytology for unsatisfactory test)** |
| --- | --- | --- | --- | --- | --- |
| Weighted average cost of a consultation | See Supplementary Table 1 | $39.38 | 0.767^a^ | $30.20 | $39.38 |
| Pap test | 73053, 73057 | $19.60 | - | $19.60 | $19.60 |
| Initiation of patient episode (PEI)^b^ | 73922 | $8.25 | - | $8.25 | $8.25 |
| **Total Cost** |  |  |  | **$58.05** | **$67.23** |

*^a^ This weighting only applied on the consultation of initial screening test*

*^b^ PEI is a Medicare benefit pay for the services provided by laboratory which receives the original request to examine the cytology test outcome.*

## Costs related to results/referral following abnormal cytology

The model assumed that any abnormal outcome of a cytology test result would prompt a medical consultation for the purpose of communicating the test results and discussing further management to the patient (when immediate referral for colposcopy is required, this visit would also involve providing a referral to a specialist). A single cost of medical consultation $39.38 was applied in the model under the assumption that this is the only reason for medical consultation.

## Costs of diagnostic procedures

The diagnostic procedures following an abnormal cytology test result in the model involved colposcopy, and punch-biopsy if indicated. Supplementary Table 4 contains the summary of the costs of diagnostic procedures. The cost of diagnosing with colposcopy only includes a cost of specialist consultant and a cost of colposcopy procedure. The cost of a colposcopy with biopsy comprised of costs for a specialist consultation, a biopsy procedure, weighted histopathology procedure cost (weightings were derived from 2010 MBS use data) and a discounted colposcopy procedure. Because both the colposcopy and biopsy procedures are performed at the same time, according to MBS Schedule note T8.2 Multiple Operations Rule[1], the cost of colposcopy procedure is discounted by half. It is assumed that all colposcopies conducted for the purpose of abnormal cytology result diagnosis are performed in specialist’s room without anaesthesia.

Based on the analysis of colposcopy data provided by Dr Jeffrey Tan, The Royal Women’s Hospital, Melbourne, the model assumed that a cytology test would be performed for 85% of colposcopies.

**Supplementary Table 4. Cost of diagnostic procedures.**

| **Components** | | **MBS item** | **Unit cost (100% Schedule)** | **Proportion of patients** | **Cost** |
| --- | --- | --- | --- | --- | --- |
| **Cost of colposcopy without biopsy** | | |  |  |  |
|  | Specialist consultation | 104 | $82.30 | 1 | $82.30 |
|  | Colposcopy | 35614 | $61.45 | 1 | $61.45 |
|  | Pap test | 73053, 73057 | $19.60 | 0.8475 | $16.61 |
|  | PEI ^a^ | 73922 | $8.25 | 0.8475 | $6.99 |
|  | **Total** |  |  |  | **$167.35** |
|  |  |  |  |  |  |
| **Cost of colposcopy with biopsy** | | |  |  |  |
|  | Specialist consultation | 104 | $82.30 | 1 | $82.30 |
|  | Colposcopy ^b^ | 35614 | $30.73 | 1 | $30.73 |
|  | Biopsy | 35608 | $61.55 | 1 | $61.55 |
|  | Histopathology ^c^ | 72823 | $97.80 | 0.6549 | $64.05 |
|  |  | 72824 | $142.30 | 0.3451 | $49.11 |
|  | PEI ^d^ | 73926 | $8.25 | 1 | $8.25 |
|  | Pap test | 73053, 73057 | $19.60 | 0.8475 | $16.61 |
|  | **Total** |  |  |  | **$312.59** |

^a^ This PEI cost is associated with the laboratory services for cytology sample examination. Because only 84.75% of the patient will have a cytology test, therefore a weighting of 0.8475 was applied.

^b^ Cost discounted by half according to MBS multiple operation Rule, Note T8.2

^c^ The proportions were informed by 2010 MBS use data (525,027 services for MBS item 723823; 276,666 services for MBS item 72824)

^d^ This PEI cost is associated with the laboratory services for the examinations for biopsy sample and cytology sample. Since all patients will have a biopsy sample taken and examined cost, no weighting was applied to this cost.

## Cost of precancerous lesion treatment

The cost of treating cervical intraepithelial neoplasm grade 2 or 3 (CIN2/3) lesions was composed of the weighted-average of the cost of ablation treatments (including laser therapy without hospitalisation and diathermy with hospitalisation), excision treatments (loop excision without hospitalisation and cone biopsy with hospitalisation) and hysterectomy. The summary of the cost calculation is shown in Supplementary Table 5. We assumed 5% of patient with CIN2/3 will be treated with hysterectomy (informed by the analysis of the colposcopy and treatment data provided by Dr Jeffrey Tan, The Royal Women’s Hospital, and Melbourne). The Royal Women’s Hospital is a tertiary referral centre in Melbourne for more complex cases, therefore this proportion derived from the dataset might be higher than the average rate among all patient diagnosed with CIN2/3. Based on the MBS use data in 2010, obtained from the Medicare online database, we assumed 15% and 80% of women with CIN2/3 will be treated with ablation treatment and excision treatment, respectively.

**Supplementary Table 5. Cost of precancerous lesion treatment**

| **Components** | | | | | **MBS / DRG item** | **Unit cost (100% Schedule)** | **Proportion of patients** | **Cost** |
| --- | --- | --- | --- | --- | --- | --- | --- | --- |
| **Ablation therapy (15% of total CIN2/3 treatment)** | | | | | | |  |  |
|  | *Laser Therapy (50% of ablation therapy)* | | | | | | | |
|  |  | Specialist consult | | | 104 | $82.30 | 1 | $82.30 |
|  |  | Colposcopy ^a^ | | | 35614 | $30.73 | 1 | $30.73 |
|  |  | Laser therapy for 1 anatomical site ^b^ | | | 35539 | $262.60 | 0.96 | $252.50 |
|  |  | Laser therapy for 2 or anatomical sites ^b^ | | | 35542 | $307.50 | 0.04 | $11.83 |
|  |  | Total cost of laser therapy | | | | | | $377.35 |
|  | *Diathermy (50% of ablation therapy)* | | | | | | |  |
|  |  | Specialist consult | | | 104 | $82.30 | 1 | $82.30 |
|  |  | Diathermy, public hospital | | | DRG N09Z | $2,116.00 | 1 | $2,116.00 |
|  |  | Total cost of diathermy | | |  |  |  | $2,198.30 |
|  | ***Total cost of ablation therapy*** | | | |  |  |  | ***$1,287.83*** |
|  | | | | | |  |  |  |
| **Excision therapy (80% of total CIN2/3 treatment)** | | | | | | | | |
|  | *LEEP no hospitalisation (85.5% of excision therapy)* | | | | | | | |
|  |  | Specialist consult | | | 104 | $82.30 | 1 | $82.30 |
|  |  | Cervix, LLETZ together with colposcopy ^c^ | | | 35647 | $195.95 | 0.94 | $184.77 |
|  |  | Cervix, LLETZ, in conjunction with ablative treatment of additional areas of intraepithelial change ^c^ | | | 35648 | $306.60 | 0.06 | $17.50 |
|  |  | Histopathology | | | 72830 (level 5 complexity) | $276.00 | 1 | $276.00 |
|  |  | Initiation of patient episode | | | 73926 | $8.25 | 1 | $8.25 |
|  |  | Total cost of LEEP | | |  |  |  | $568.81 |
|  | *Cone biopsy (14.5% of excision therapy)* | | | | | | | |
|  |  | Specialist consult | | | 104 | $82.30 | 1 | $82.30 |
|  |  | Hospitalisation, public hospital | | | DRG N09Z | $2,116.00 | 1 | $2,116.00 |
|  |  | Total cost of cone biopsy | | | | |  | $2,198.30 |
| ***Total cost of excision therapy*** | | | | | | | | ***$804.87*** |
|  | | |  |  | | |  |  |
| **Hysterectomy for non-cervical cancer related reason (5% of total CIN2/3 treatment)** | | | | | | | | |
| Specialist consult | | | | | 104 | $82.30 | 1 | $82.30 |
| Hysterectomy for non-malignancy | | | | | DRG N04Z | $8,441.00 | 1 | $8,441.00 |
| ***Total cost of hysterectomy*** | | | | |  |  |  | ***$8,523.30*** |
|  | | |  |  |  |  |  |  |
| **Total weighted average cost of treatment for CIN2/3** | | | | | | | | **$1,263.23** |

*^a^ MBS multiple operation rule Note T8.2*

*^b^ Proportion breakdown were calculated according 2010 MBS utilisation data (1,050 services for MBS item 35539, 42 services for MBS item 35542 )*

*^c^ Proportion breakdown were calculated according 2010 MBS utilisation data (6,643 services for MBS item 35647, 402 services for MBS item 3564 )*

*DRG: Diagnosis-related group; LEEP: Large electrical excision procedure; LLETZ: Large loop excision of transformation zone*

## Cost of post-treatment follow-up

The post-treatment follow-up was modelled according to the management pathway in the 2005 National Health and Medical research Council (NHMRC) Guidelines, which involve managing patients treated for CIN2/3 at 4-6 months post-treatment with cytology and colposcopy, and then repeat annual screening, using HPV testing and cytology, commencing 12 months after treatment until the women test negative for both tests in two consecutive occasions.[5,8] The cost of follow-up within 4-6 months after treatment of CIN2/3 with cytology and colposcopy is $130.65. The cost of managing women previously treated for CIN2/3 with test-of-cure management is $131.23 for each visit. The breakdown of the aggregated costs for post-treatment follow-up is summarised in Supplementary Table 6.

**Supplementary Table 6. Cost post-treatment follow-up after CIN2/3 treatment**

| **Components** | | **MBS item** | **Unit cost (100% Schedule)** | **Proportion of patients** | **Cost** |
| --- | --- | --- | --- | --- | --- |
| **Cost of 4-6 months post-treatment follow-up after CIN2/3 treatment** | | | | | |
|  | Specialist consult | 105 | $41.35 | 1 | $41.35 |
|  | PEI | 73922 | $8.25 | 1 | $8.25 |
|  | Pap test | 73053, 73057 | $19.60 | 1 | $19.60 |
|  | Colposcopy | 35614 | $61.45 | 1 | $61.45 |
| ***Total*** | |  |  |  | ***$130.65*** |
|  |  |  |  |  |  |
| **Cost of managing women with post-treatment test-of-cure procedures (per visit)** | | | | | |
|  | Weighted average cost of a consultation | See Supplementary Table 1 | $39.38 | 1 | $39.38 |
|  | PEI | 73922 | $8.25 | 1 | $8.25 |
|  | Pap test | 73053, 73057 | $19.60 | 1 | $19.60 |
|  | HPV test | 69418 | $64.00 | 1 | $64.00 |
| ***Total*** | |  |  |  | ***$131.23*** |

## Cost of cancer treatment and work-up by stage and disease extent

There are no direct data available for the stage-specific treatment or work-up costs for cervical cancer in Australia. The likely treatment practices by International Federation of Gynecology and Obsterics (FIGO) disease stage was informed by consensus expert opinion of the Advisory Panel of MSAC report 1122 and report 39 [6,7]. Distribution of cancer by FIGO stage at the time of diagnosis was based on data provided by the Queensland Gynaecological Cancer Centre and the Royal Women’s Hospital, Melbourne.

The model was structured to model the natural history of cervical cancer by extent of disease because the available calibration data was categorised by extent of disease. Therefore, the data by FIGO stage were grouped to broadly represent the extent of disease categories. The summary of stage-specific work-up costs for cervical cancer diagnosis and cancer treatment are shown in Supplementary Table 7.

Due to the absence of data on the distribution of treatment cost over time, all cancer treatment costs were applied as once-off costs in the year of diagnosis. Terminal care costs were applied in the year a patient dies from cervical cancer.

**Supplementary Table 7. Summary work-up and treatment by FIGO stage and disease extension**

| **Disease extent** | **Distribution of FIGO cancer stage in disease extent category ^a^** | **Cancer work-up costs** | | **Cancer treatment cost** | | |
| --- | --- | --- | --- | --- | --- | --- |
|  |  | **Stage-specific costs ^b^** | **Proportion weighted costs** | **Stage-specific costs ^b^** | | **Proportion weighted costs** |
| **Localised cervical cancer** | | |  |  | |  |
| FIGO Ia NOS | 2.0% | $1,693.88 | $34.68 | $7,330.55 | | $150.07 |
| FIGO Ia1 | 30.4% | $1,693.88 | $514.83 | $7,330.55 | | $2,228.03 |
| FIGO Ia2 | 8.2% | $1,693.88 | $138.71 | $13,164.32 | | $1,078.02 |
| FIGO Ib NOS | 12.8% | $1,693.88 | $216.07 | $16,270.50 | | $2,075.45 |
| FIGO Ib1 | 46.6% | $1,693.88 | $789.59 | $16,270.50 | | $7,584.36 |
| ***Total*** | ***100.0%*** |  | ***$1,693.88*** |  | | ***$13,115.93*** |
|  |  |  |  |  | |  |
| **Locally advanced / regional cervical cancer** | | | | | | |
| FIGO Ib2 | 21.6% | $2,145.02 | $462.65 | $31,578.50 | $6,811.05 | |
| FIGO IIa | 9.6% | $1,816.87 | $174.56 | $30,698.70 | $2,949.48 | |
| FIGO IIb | 33.7% | $1,816.87 | $612.75 | $32,458.30 | $10,946.72 | |
| FIGO IIIa | 6.7% | $1,816.87 | $121.12 | $32,458.30 | $2,163.89 | |
| FIGO IIIb | 21.6% | $1,816.87 | $391.87 | $32,458.30 | $7,000.81 | |
| FIGO IIIc | 0.8% | $1,816.87 | $14.25 | $32,458.30 | $254.57 | |
| FIGO IVa | 6.1% | $1,864.85 | $113.35 | $31,615.05 | $1,921.70 | |
| ***Total*** | ***100.0%*** |  | ***$1,890.57*** |  | ***$32,048.22*** | |
|  |  |  |  |  |  | |
| **Distant cervical cancer** | | | |  |  | |
| FIGO IVb | 100.0% | $1,864.85 | $1,864.85 | $24,250.30 | $24,250.30 | |
| ***Total*** | ***100.0%*** |  | ***$1,864.85*** |  | ***$24,250.30*** | |

*NOS: Not otherwise specified*

^a^ Based on data from Queensland Gynaecological Cancer Centre and Royal Women’s Hospital, Melbourne

^b^ See Supplementary Table 8 for the detail calculation of aggregated cost for stage-specific cervical cancer work-up and treatment by FIGO stage

**Supplementary Table 8.Summary stage-specific cervical cancer work-up and treatment costs by FIGO stage.**

| **Treatments** | | **Unit Cost** | **Source** | **Stage-specific treatment proportion among patients diagnose with cervical cancer** | | | | | | | | | | |
| --- | --- | --- | --- | --- | --- | --- | --- | --- | --- | --- | --- | --- | --- | --- |
|  |  |  |  | **Ia1** | **Ia2** | **Ib1** | **Ib2** | | **IIa** | **IIb** | **IIIa** | **IIIb** | **IVa** | **IVb** |
| **Cervical cancer work-up ^a^** | | | | | | | | | | | | | | |
| Colposcopy | | $213.68 | Supplementary Table 5 (without specialist consultation) | 100% | 100% | 100% | 100% | 90% | | 90% | 90% | 90% | 90% | 90% |
| Chest x-ray | | $47.15 | MBS 58503 | 100% | 100% | 100% | 100% | 100% | | 100% | 100% | 100% | 100% | 100% |
| CT scan | | $480.05 | MBS 56507 | 100% | 100% | 100% | 100% | 100% | | 100% | 100% | 100% | 100% | 100% |
| PET scan | | $953.00 | MBS 61529 | 100% | 100% | 100% | 100% | 100% | | 100% | 100% | 100% | 100% | 100% |
| MRI ^b^ | | $451.14 | MBS 63470 & 63473 | - | - | - | 100% | - | | - | - | - | - | - |
| Bone scan | | $479.80 | MBS 61421 | - | - | - | - | - | | - | - | - | 10% | 10% |
| Cystoscopy | | $160.40 | MBS 36812 | - | - | - | - | 90% | | 90% | 90% | 90% | 90% | 90% |
|  |  |  |  |  |  |  |  |  | |  |  |  |  |  |
| **Stage-specific work-up costs** | | |  | **$1,693.88** | **$1,693.88** | **$1,693.88** | **$2,145.02** | **$1,816.87** | | **$1,816.87** | **$1,816.87** | **$1,816.87** | **$1,864.85** | **$1,864.85** |
|  |  |  |  |  | ` |  |  |  | |  |  |  |  |  |
| **Cervical cancer treatment ^c^** | | |  |  |  |  |  |  | |  |  |  |  |  |
| Surgery alone ^d^ | |  |  |  |  |  |  |  | |  |  |  |  |  |
|  | Conisation | $2,198.30 | Supplementary Table 9 | 25% | - | - | - | - | | - | - | - | - | - |
|  | Hysterectomy | $9,041.30 | Supplementary Table 9 | 75% | 50% | - | - | - | | - | - | - | - | - |
|  | Radical hysterectomy | $14,862.30 | Supplementary Table 9 | - | 50% | 85% | 5% | 10% | | - | - | - | - | - |
|  | Exenteration | $15,593.30 | Supplementary Table 9 | - | - | - | - | - | | - | - | - | 5% | - |
| Radiatheraphy alone | | $24,250. 30 | Supplementary Table 10 | - | - | 15% | - | - | | - | - | - | - | 100% |
| Adjuvant radiotheraphy | | $24,250. 30 | Supplementary Table 10 | - | 5% | - | - | - | | - | - | - | - | - |
| Chemo-radiation | | $32,458.30 | Supplementary Table 10 | - | - | - | 95% | 90% | | 100% | 100% | 100% | 95% | - |
|  |  |  |  |  |  |  |  |  | |  |  |  |  |  |
| **Stage-specific treatment cost** | | | | **$7,330.55** | **$13,164.32** | **$16,270.50** | **$31,578.50** | **$30,698.70** | | **$32,458.30** | **$32,458.30** | **$32,458.30** | **$31,615.05** | **$24,250.30** |

^a^ Work-ups proportions from N. Hacker, personal communication.

^b^ The cost of MRI contains 78.6% of MBS item 63470 (unit cost $403.20) and 21.4% of MBS item 63473 (unit cost $627.20). The weights were informed by 2010 MBS utilisation data.

^c^ Treatment proportions form Prof.Ian Hammond (via L. Farrell).

^d^ Stage Ia1 surgery = 25% conisation (DRG N09Z) 75% hysterectomy (DGR N03A/B) Neville Hacker, personal communication).

We assumed surgical management for cervical cancer treatment comprises conisation, simple hysterectomy, radical hysterectomy and exenteration.[6,7] The current diagnosis-related groups (DRGs) do not distinguish between a simple and radical hysterectomy, therefore, we assumed that a simple hysterectomy has a cost equivalent to a hysterectomy without complication, while a radical hysterectomy is assumed to have a cost equivalent to a hysterectomy with complication. The detailed calculations for the cost of surgical management are shown in Supplementary Table 9.

**Supplementary Table 9. Cost of surgical managements for cervical cancer treatment**

| **Components** | | **MBS/ DRG item** | **Unit cost** | **proportion of patients** | **Cost** |
| --- | --- | --- | --- | --- | --- |
| **Cost of conisation** | | | | | |
|  | Specialist consult | 104 | $82.30 | 1 | $82.30 |
|  | Conisation, public hospital | DRG N09Z | $2,116.00 | 1 | $2,116.00 |
|  | ***Total*** |  |  |  | ***$2,198.30*** |
|  |  |  |  |  |  |
| **Cost of simple hysterectomy (assume as for hysterectomy without complication)** | | | | | |
|  | Specialist consult | 104 | $82.30 | 1 | $82.30 |
|  | Uterine, Adnexa Procedure for Non-ovarian/Adnexal Malignancy without complication | DRG N03B | $8,959.00 | 1 | $8,959.00 |
|  | ***Total*** |  |  |  | ***$9,041.30*** |
|  |  |  |  |  |  |
| **Cost of radical hysterectomy including nodes (assume as for hysterectomy with complication)** | | | | | |
|  | Specialist consult | 104 | $82.30 | 1 | $82.30 |
|  | Uterine, Adnexa Procedure for Non-ovarian/Adnexal Malignancy with complication | DRG N03A | $14,780.00 | 1 | $14,780.00 |
|  | ***Total*** |  |  |  | ***$14,862.30*** |
|  |  |  |  |  |  |
| **Cost of exenteration** | |  |  |  |  |
|  | Specialist consult | 104 | $82.30 | 1 | $82.30 |
|  | Pelvic evisceration & radical vulvectomy | DRG N01Z | $15,511.00 | 1 | $15,511.00 |
|  | ***Total*** |  |  |  | ***$15,593.30*** |

Non-surgical management comprises primary radiotherapy, adjuvant radiotherapy and chemo-radiotherapy. The adjuvant radiotherapy was assumed to have the same cost as primary radiotherapy. See Supplementary Table 10 for the details of the cost calculation.

**Supplementary Table 10. Cost of non-surgical managements**

| **Components** | | **MBS/ DRG item** | **Unit cost** | **Units** | **Cost** |
| --- | --- | --- | --- | --- | --- |
| **Cost of radiation** | | | | | |
|  | Specialist consult | 104 | $82.30 | 1 | $82.30 |
|  | Inpatient radiation | DRG R64Z | $2,014.00 | 2 | $4,028.00 |
|  | Outpatient radiation ^a^ | DRG R64Z | $1,007.00 | 20 | $20,140.00 |
|  | ***Total*** |  |  |  | ***$24,250.30*** |
|  |  |  |  |  |  |
| **Cost of chemo-radiation** | | | | | |
|  | Specialist consult | 104 | $82.30 | 1 | $82.30 |
|  | Inpatient radiation | DRG R64Z | $2,014.00 | 2 | $4,028.00 |
|  | Outpatient radiation ^a^ | DRG R64Z | $1,007.00 | 20 | $20,140.00 |
|  | Outpatient chemotherapy ^b^ | DRG R63Z | $1,368.00 | 6 | $8,208.00 |
|  | *Total* |  |  |  | ***$32,458.30*** |

*^a^Assumed to be 50% cost of inpatient radiation.*

*^b^ Assumed to be same as cost of inpatient chemotherapy (length of stay = 1 day).*

**Reference List**

1. Australian Government Department of Health and Ageing. **MBS online: Medicare Benefits Schedule** [<http://www.mbsonline.gov.au/internet/mbsonline/publishing.nsf/Content/Medicare-Benefits-Schedule-MBS-1>]

2. Commonwealth Department of Health and Aged Care: *National Hospital Cost Data Collection: Cost report Round 13 (2009-2009).* Canberra; 2010.

3. Australian Government Department of Health and Ageing. **Pharmaceutical Benefits Scheme (PBS)** [<http://www.pbs.gov.au/pbs/home>]

4. Australian Government Medicare Australia. **Medicare Item Report** [<https://www.medicareaustralia.gov.au/statistics/mbs_item.shtml>]

5. Britt H, Miller GC, Charles J, Henderson J, Bayram C, Pan Y, Valenti L, Harrison C, O'Halloran J, Fahridin S: *General practice activity in Australia 2009–10. General practice series no. 27.Cat. no. GEP 27.* Canberra; 2010.

6. Medical Services Advisory Committee: *Human Papillomavirus Triage Test For Women With Possible or Definite Low-Grade Squamous Intraepithelial Lesions. MSAC reference 39, Assessment report.* 2009.

7. Medical Services Advisory Committee: *Automation Assisted and Liquid Based Cytology for Cervical Cancer Screening. MSAC reference 1122, Assessment report.* 2009.

8. National Health and Medical Research Council: *Screening to Prevent Cervical Cancer: Guidelines for the Management of Asymptomatic Women with Screen Detected Abnormalities.* Canberra; 2005.
